# Temperature Compensation Method Based on an Improved Firefly Algorithm Optimized Backpropagation Neural Network for Micromachined Silicon Resonant Accelerometers

**DOI:** 10.3390/mi13071054

**Published:** 2022-06-30

**Authors:** Libin Huang, Lin Jiang, Liye Zhao, Xukai Ding

**Affiliations:** 1 School of Instrument Science and Engineering, Southeast University, Nanjing 210096, China; 220203458@seu.edu.cn (L.J.); liyezhao@seu.edu.cn (L.Z.); ding.xk@seu.edu.cn (X.D.); 2 Key Laboratory of Micro-Inertial Instruments and Advanced Navigation Technology, Ministry of Education, Nanjing 210096, China

**Keywords:** micromachined silicon resonant accelerometer, temperature compensation, neural network, firefly algorithm

## Abstract

The output of the micromachined silicon resonant accelerometer (MSRA) is prone to drift in a temperature-changing environment. Therefore, it is crucial to adopt an appropriate suppression method for temperature error to improve the performance of the accelerometer. In this study, an improved firefly algorithm-backpropagation (IFA-BP) neural network is proposed in order to realize temperature compensation. IFA can improve a BP neural network’s convergence accuracy and robustness in the training process by optimizing the initial weights and thresholds of the BP neural network. Additionally, zero-bias experiments at room temperature and full-temperature experiments were conducted on the MSRA, and the reproducible experimental data were used to train and evaluate the temperature compensation model. Compared with the firefly algorithm-backpropagation (FA-BP) neural network, it was proven that the IFA-BP neural network model has a better temperature compensation performance. The experimental results of the zero-bias experiment at room temperature indicated that the stability of the zero-bias was improved by more than an order of magnitude after compensation by the IFA-BP neural network temperature compensation model. The results of the full-temperature experiment indicated that in the temperature range of −40 °C~60 °C, the variation of the scale factor at full temperature improved by more than 70 times, and the variation of the bias at full temperature improved by around three orders of magnitude.

## 1. Introduction

Micromachined silicon resonant accelerometers have the advantages of small size, low power consumption, mass production, and quasi-digitalization [1,2]. They have been widely used in aerospace and Earth exploration fields [3,4]. Due to the influence of the materials and fabrication, the output of the MSRA is prone to drift in a temperature-changing environment. The influence of temperature on the accelerometer is mainly reflected in the following aspects: firstly, the Young’s modulus of the silicon will change with temperature [5]; secondly, the mismatched thermal expansion coefficient of silicon and the base material will create thermal stress in the resonator; thirdly, the fabricating and packaging process will lead to the generation of residual thermal stress [6,7,8]. Temperature drift is one of the key factors limiting further improvements in the accuracy of MSRAs, and it needs to be suppressed [9]. At present, the common temperature drift suppression methods mainly include temperature control systems, structural optimization, and temperature compensation models.

The temperature control system can make an accelerometer operate at a constant temperature, which effectively avoids the influence of temperature change. In general, a temperature chamber or an on-chip heating electrode is used to keep the temperature stable; therefore, the temperature control system can theoretically eliminate the influence of temperature across a wide working range. However, the temperature chamber will greatly increase the volume and power of the system, and the on-chip heating electrode method is complicated to control and may introduce additional noise [10,11,12,13]. Structural optimization mainly reduces the influence of thermal stress by designing a special structure, such as a differential structure or a stress isolation frame [14,15,16,17]. The authors of [18] proposed a differential resonant accelerometer with temperature compensation, in which resonant beams were fixed by a single anchor to eliminate the frequency drift caused by external and package stress. In [19], the authors reported an MSRA embedded in an isolation frame with stress-insensitive anchors, which could protect the resonant beams from thermal stress along the sensitive axis. The method of structural optimization can theoretically reduce the influence of thermal stress effectively. However, due to the limitations of processing technology, it is difficult to achieve complete symmetry in the differential structure, which means that the common-mode error cannot be completely eliminated.

Temperature drift compensation based on a software algorithm is an efficient method for suppressing temperature drift. Establishing a temperature compensation model can save numerous hardware resources and has great flexibility [20]. The polynomial compensation model is a commonly used temperature compensation model. The authors of [21] used temperature resonators to measure the temperature and established a polynomial temperature compensation model to compensate the frequency output of the accelerometer. The experimental results showed that the temperature coefficients of the bias and scale factor reduced from 3.1 $mg/{}^{\circ }C$ and 778 $ppm/{}^{\circ }C$ to 0.05 $mg/{}^{\circ }C$ and −9.4 $ppm/{}^{\circ }C$ after compensation. The authors of [22] proposed a method of self-calibration of the temperature difference ratio and established a temperature compensation model for a resonant accelerometer. A least-squares method which performed regression analysis on the accelerometer’s frequency and temperature was applied to obtain the model parameters. The experimental results showed that the frequency drift of the two resonators of the accelerometer reduced from 43.16 $ppm/{}^{\circ }C$ to 0.83 $ppm/{}^{\circ }C$ after compensation.

In recent years, with the development of machine learning technology, neural networks have been widely applied in many fields [23,24,25], and accelerometer temperature compensation models established by neural networks have also begun to be researched. The authors of [26] proposed a temperature compensation method based on the BP neural network and combined it with the genetic algorithm to optimize the BP neural network model. The experimental results showed that the maximum error of the accelerometer’s output was 0.017%, which is 173 times better than the traditional polynomial fit over the temperature range from −10 ${}^{\circ }C$ to 60 ${}^{\circ }C$. In [27], the authors proposed a fusion algorithm of EMD (empirical mode decomposition) + wavelet thresholding + GA (genetic algorithm) BP temperature compensation to improve the accuracy of a high-G accelerometer. The experimental results showed that the random walk and zero-bias stability of the accelerometer changed from 1712.66 $g/h/\sqrt{Hz}$ and 49275 g/h to 79.15 $g/h/\sqrt{Hz}$ and 774.7 g/h, respectively. The authors of [28] proposed an RBF-NN + GA + KF (radial basis function neural network + genetic algorithm + Kalman filter) fusion algorithm to compensate for the temperature drift of an accelerometer. With this method, the acceleration random drift was reduced from 17130 $g/h/\sqrt{Hz}$ to 765.3 $g/h/\sqrt{Hz}$, and the bias stability reduced from 4720 g/h to 57.27 g/h.

To further improve the performance of the MSRA developed in the laboratory, a method for temperature compensation based on an IFA-BP neural network model is proposed in this study. It optimizes the initial weights and thresholds of the BP neural network by taking advantage of the optimization ability of IFA and then applies the obtained neural network model to perform compensation for the accelerometer to improve the temperature performance of the accelerometer. Finally, a temperature experimental system was established to verify the effect of the IFA-BP neural network compensation model. The results showed that the stability of the zero-bias improved by more than 10 times after compensation in the zero-bias experiment at room temperature. The full-temperature experiment indicated that in the temperature range of −40 °C~60 °C, the variation of the scale factor at full temperature improved by more than 70 times, and the variation of the bias at full-temperature improved by around 1000 times after compensation.

## 2. Establishing the IFA-BP Neural Network Compensation Model

### 2.1. BP Neural Network

**Figure 1:**
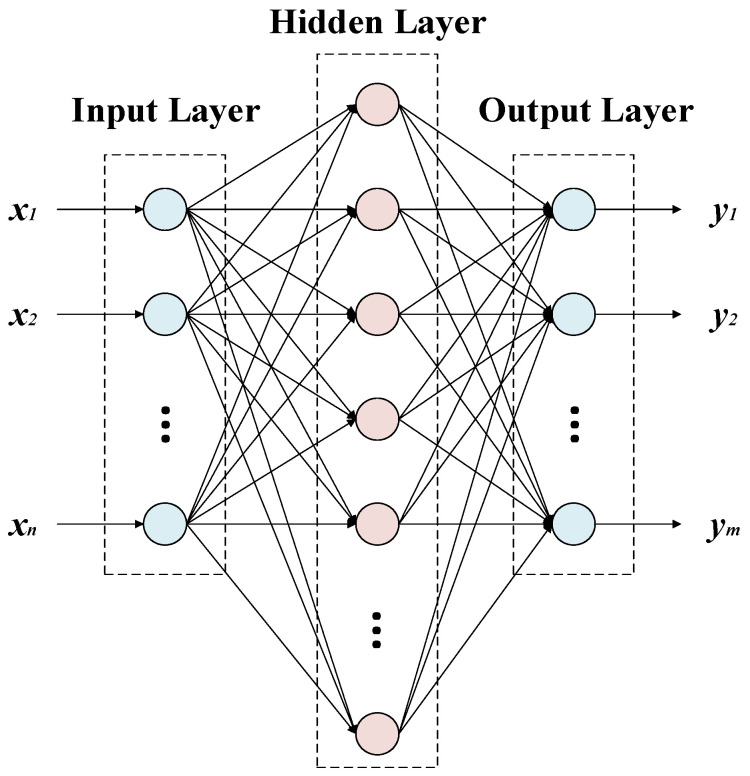
Three-layer neural network.

The BP neural network is a multilayer feedforward neural network based on error backpropagation, and its learning process includes forward signal propagation and error backpropagation. The BP neural network is composed of an input layer, a hidden layer, and an output layer. When the number of hidden layer neurons is appropriately determined, a three-layer neural network with a single hidden layer can achieve the approximation of an arbitrary nonlinear function [29]. For problems with low complexity, a neural network with one hidden layer is sufficient, and an excessive number of hidden layers may lead to difficulties in convergence. The structure of the three-layer BP neural network is shown in Figure 1. The BP neural network does not need the relational expression between the input and output, and only needs to be trained with a large amount of data to obtain a high-precision model. Therefore, it has the advantages of strong self-adaptation and strong learning and it has been widely used in many fields.

To conduct the training of the BP neural network, the topology of the neural network should first be established, which involves determining the number of hidden layers and the number of neurons in each layer. The number of input neurons and output neurons is determined by the application. In this study, the input data are the frequency difference and the temperature, so the number of input neurons is m=2. The output data are the corrected acceleration values, so the number of output neurons is n=1. The number of hidden neurons can be determined by the following equation [30]:

(1) $$l=\sqrt{m+n}+c$$

where c is a constant between 0 and 10.

After establishing the topology of the neural network, the weights and thresholds of the neural network need to be initialized. By inputting the signal $X=x_{1},x_{2},\cdots ,x_{m}$ into the neural network through the input layer, the output is obtained [31]:

(2) $$y_{n}=\varphi \left(\sum_{i=1}^{l}w_{li}\sigma \left(\sum_{j=1}^{m}w_{mj}x_{j}+b_{j}\right)+b_{n}\right)$$

where σ is the activation function of the hidden layer, φ is the activation function of the output layer; $w_{mj}$ is the weight assigned by the input layer to the $j_{th}$ neuron of the hidden layer, $w_{li}$ is the weight from the hidden layer to the $i_{th}$ neuron of the output layer, $b_{i}$ is the threshold of the $i_{th}$ neuron of the hidden layer, and $b_{n}$ is the threshold of the $n_{th}$ neuron of the output layer. The output of the neural network is compared with the expected output to obtain the network error function.

(3) $$C=\frac{1}{2n}\sum_{k=1}^{n}\|\bar{y_{k}}-y_{k}{\|}^{2}$$

where $\bar{y_{k}}$ is the expected output. The weights and thresholds of the neural network are corrected backward by finding the partial derivatives of the error function and by using a gradient descent until the error or the number of iterations reaches the set value. However, BP neural networks have inherent disadvantages, such as low convergence accuracy and low robustness. Some researchers have proposed the use of modern optimization algorithms to improve the performance of BP neural networks. The firefly algorithm is one of the modern optimization algorithms which simulates the luminous characteristics and attraction behavior of fireflies and has the advantages of a simple structure, few adjustment parameters, and excellent search capability.

### 2.2. Firefly Algorithm

#### 2.2.1. Standard Firefly Algorithm

The firefly algorithm was first proposed by Xin-She Yang in 2008 [32] and is a heuristic algorithm derived from the behavior of fireflies in nature. The basic principle of FA is that each firefly can emit light, and the intensity of its brightness is related to its position. Fireflies with high brightness will attract fireflies with less brightness, and the greater the intensity of brightness, the greater the attraction. By updating the position of the fireflies, we gradually find the position with the highest intensity of brightness. In FA, the intensity of brightness is the value of the objective function, and the position is the feasible solution to the problem to be solved.

The method randomly initialized n fireflies in the *D*-dimensional space, with each firefly positioned at $X=\left(x_{1},x_{2},\cdots ,x_{D}\right)$. Therefore, the attractiveness of a firefly is [33]:

(4) $$\beta =\beta_{0}e^{-\gamma r_{ij}^{2}}$$

where $\beta_{0}$ is the initial attractiveness, γ is the light absorption coefficient, and $r_{ij}$ is the Euclidean distance between the firefly $X_{i}$ and firefly $X_{j}$. Each firefly will move toward all fireflies whose brightness is greater than its own, and its position is updated by the equation:

(5) $$X_{i}\left(n+1\right)=X_{i}\left(n\right)+\beta_{0}e^{-\gamma r_{ij}^{2}}\left(X_{j}\left(n\right)-X_{i}\left(n\right)\right)+\alpha_{0}\left(\xi -\frac{1}{2}\right)$$

where n is the number of iterations, $\alpha_{0}$ is the step size factor, and ξ is a random number subject to uniform distribution on [0,1].

#### 2.2.2. Improved Firefly Algorithm

The FA has been used in many fields since it was proposed, but it has disadvantages; for example, it easily falls into bad local minima and has possible oscillations in the later iterations. To address these problems, an improved firefly algorithm (IFA) was proposed to further improve the optimal finding ability and stability of the FA.

1. Improvement of the step size strategy

In the standard FA, the step size is constant during the iterations. If the chosen step size is too large, the algorithm can quickly move to the optimum at the beginning of the iterations, which makes the algorithm have a strong global search capability; however, at the end of the iterations, the optimum may be skipped or the iteration may oscillate due to the large step size, which greatly reduces the accuracy of the algorithm. If the chosen step size is too small, it can make the algorithm approach the local optimum more accurately in the later iterations; however, in the early iterations, it will lead to slow convergence and reduce the global search capability. To balance the ability for global search and local search, an adaptive step size update formula was designed using a nonlinear function. The step size is calculated as follows:

(6) $$\alpha \left(n+1\right)=k\cdot \alpha \left(n\right)\cdot e^{\frac{n}{n-maxgen}}$$

where k is the adjustment factor and maxgen is the maximum number of iterations. According to the formula, the value of the step size decreases with an increase in the number of iterations. At the beginning of the iterations, a larger step size can make the algorithm’s global search capability stronger and improve the iteration efficiency, whereas at the end of the iteration, a smaller step size can enhance the local search capability of the algorithm and improve the optimization accuracy.

2. Improvement of the best firefly

According to the FA, each firefly is attracted to the firefly with the greatest brightness, which makes the position of the best firefly greatly affect the algorithm’s search process. For instance, if the best firefly is near the bad local minima, it is possible to make the algorithm converge to it. To update the position of the best firefly, the Metropolis criterion in the simulated annealing algorithm [34] combined with mutations is introduced. The Metropolis criterion can be expressed as follows: when the system is subjected to a perturbation that generates a new value $X^{\prime }$ as well as a new objective function value $C^{\prime }$, the system calculates the acceptance probability $P_{i}$ according to the Metropolis criterion to determine whether to update the new value. The calculation formula is as follows:

(7) $$P_{i}=\{\begin{array}{cc} 1, & C^{\prime }<C\\ \exp(\frac{C-C^{\prime }}{T}), & C^{\prime }\geq C \end{array}$$

where T is the temperature of the simulated annealing algorithm, relative to the number of iterations. 

If the objective function value of the new value is better, the new value is received with a probability of 1; otherwise, if the objective function value of the new value is worse than the original value, the new value is received with a probability of $\exp(\left(C-C^{\prime }\right)/T)$. A worse value can be received with the probability calculated by the Metropolis criterion, which enhances the stability of the algorithm and provides the opportunity to jump out of the bad local minima. The temperature in the simulated annealing algorithm gradually decreases with the number of iterations, which means the probability of accepting the worse value decreases in the later iterations. In order to apply the Metropolis criterion to the firefly algorithm, some modifications to the original formulation are required.

(8) T=ε⋅(maxgen−n)

where ε is the correction factor. Meanwhile, in order to perturb the best firefly, combining the mutations and balancing the convergence speed and accuracy, the following variational perturbation formula is used to perturb the firefly’s position.

(9) $$\Delta x=\cos\left(\frac{\pi }{2}\cdot \frac{n}{maxgen}\right)\cdot \left(\xi -\frac{1}{2}\right)\cdot s$$

where ξ is a random number in the range [0,1] and s is the width of the definition domain.

The variational perturbation formula also decreases nonlinearly with an increase in the number of iterations, avoiding or attenuating the oscillations that may result from a fixed perturbation. Thus, the position of the best firefly subject to perturbation is updated as:

(10) $$X_{B}^{\prime }=X_{B}+\Delta X$$

where $\Delta X=\left(\Delta x_{1},\Delta x_{2},\cdots ,\Delta x_{D}\right)$ is the perturbation matrix.

3. Improvement of the firefly position update strategy

In the FA, the fireflies move toward all fireflies with greater brightness, which inevitably leads to rapid convergence of the firefly population. However, if the firefly population gathers at a bad position prematurely, the search ability of the algorithm decreases rapidly, which makes it difficult to jump out of the bad local minima. The position update strategy of fireflies is improved by randomly selecting only several individuals from the firefly population, so that the fireflies only move toward selected fireflies with greater brightness. In addition, the fireflies may be out of the search space after updating the position, and the positions of fireflies that are out of the search range are corrected according to the following equation:

(11) $$x_{i}=\{\begin{array}{cc} x_{\min}, & x_{i}<x_{\min}\\ x_{\max}, & x_{i}>x_{\max} \end{array}$$

where $x_{\min}$ and $x_{\max}$ are the boundary values of the search space.

#### 2.2.3. Simulation Analysis of Optimization Algorithm Based on Test Functions

**Figure 2:**
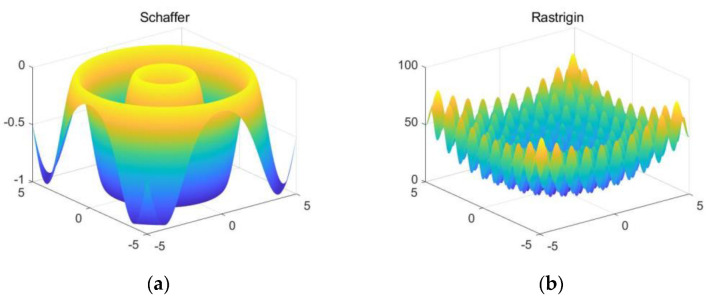
Images of the two test functions: (**a**) Schaffer function; (**b**) Rastrigin function.

Two test functions were selected to evaluate the performance of the proposed IFA algorithm and the standard FA, and images of the two test functions are shown in Figure 2. The population size of the two firefly algorithms is 10, the number of dimensions is 2, the number of iterations is 50, the initial step size $\alpha_{0}$ is 0.25, and the initial attractiveness $\beta_{0}$ and the light absorption γ are both set to 1.

**Figure 3:**
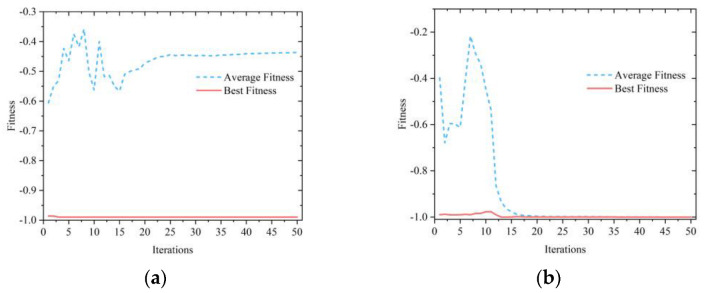
Evolutionary curve of the Schaffer function using different compensation models: (**a**) FA; (**b**) IFA.

The Schaffer function has many local minima distributed near the global minima, which can be used to evaluate the global optimization searching ability of the algorithms. The evolution curves of the two optimization algorithms for the Schaffer function are shown in Figure 3. Figure 3a shows the evolution curves of the FA. As shown in the figures, although the best fitness of the algorithm can converge to the global minima of −1, the average fitness falls to local minima around −0.45, indicating that the FA is less stable. Figure 3b shows that the IFA can make both the best fitness and the average fitness converge to the global minima at −1, which indicates that the IFA has great stability and global search capability.

**Figure 4:**
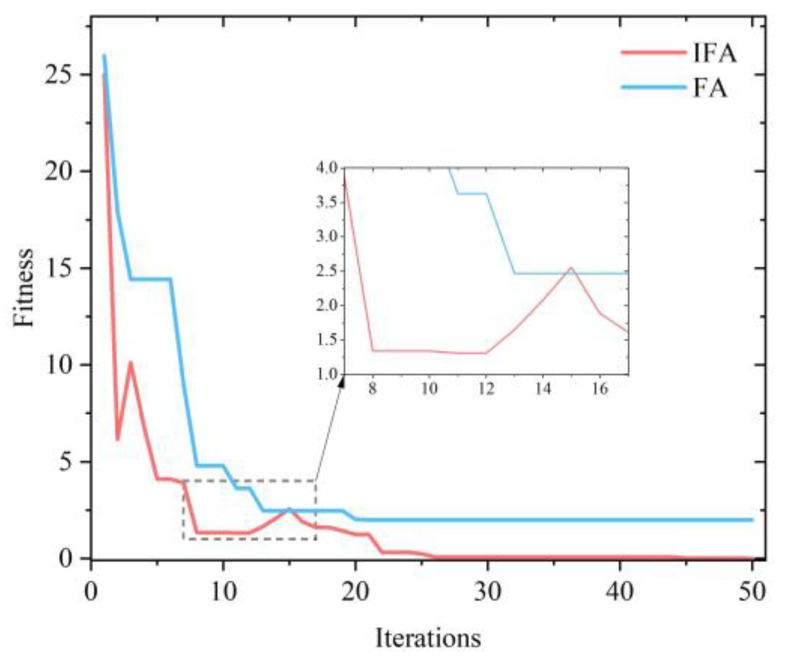
Evolutionary curve of the Rastrigin function using the FA and the IFA.

The Rastrigin function has many local minima distributed throughout the definition domain, making it easy to fall to the local minima for the optimization algorithm. Figure 4 shows the evolution curves of the two optimization algorithms for the Rastrigin function. Figure 4 shows the evolution curves of the FA and the IFA, and it can be seen that the FA falls to the local minima and converges around 2. The IFA also falls to the local minima of 1.3 at the eighth iteration, but because the improved algorithm makes it accept a worse value with a certain probability, it jumps out of the local minima of 1.3 at the 12th iteration and finally converges to the global minima of 0. This simulation results indicate that IFA has a stronger ability to jump out of the local minima compared with the FA.

### 2.3. IFA-BP Neural Network Model

**Figure 5:**
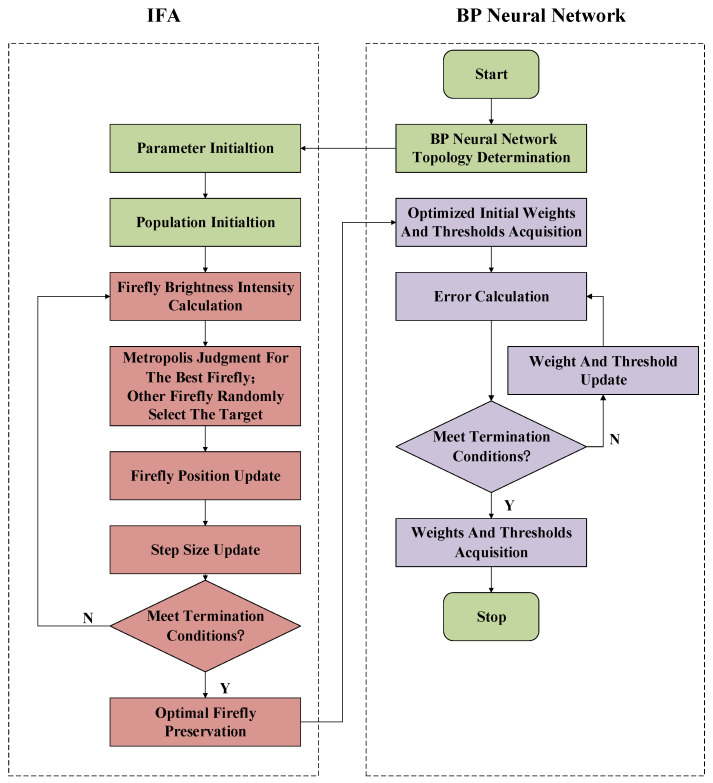
Flow chart of the IFA-BP neural network.

The selection of the initial weights and thresholds has a large impact on the training, and bad initial values may make the training too slow or even fail. The proposed IFA was used to optimize the initial weights and thresholds of the BP neural network with a better global search ability and the ability to jump out of local minima. The flow chart of the algorithm for optimizing the BP neural network by IFA is shown in Figure 5, and its main steps are as follows:

(1) Initialize the BP neural network topology and all parameters of the IFA and generate the initial population of fireflies.

(2) Calculate the brightness of the fireflies, perturb the best fireflies, and calculate the acceptance probability according to the Metropolis criterion; other fireflies randomly select the moving target and all fireflies update their positions according to the position update rule.

(3) Update the step size and determine whether the termination iteration condition is satisfied. If so, save the best firefly and jump to Step (4); otherwise, return to Step (2).

(4) Use the best firefly as the initial weights and thresholds of the BP neural network.

(5) Substitute the accelerometer output dataset into the model and calculate the error function.

(6) Determine whether the termination iteration condition has been satisfied. If it has been satisfied, save the weights and thresholds and quit training; if not, update the weights and thresholds of the neural network using the gradient descent method and return to Step (5).

## 3. Experiments and Results

### 3.1. Experiments

**Figure 6:**
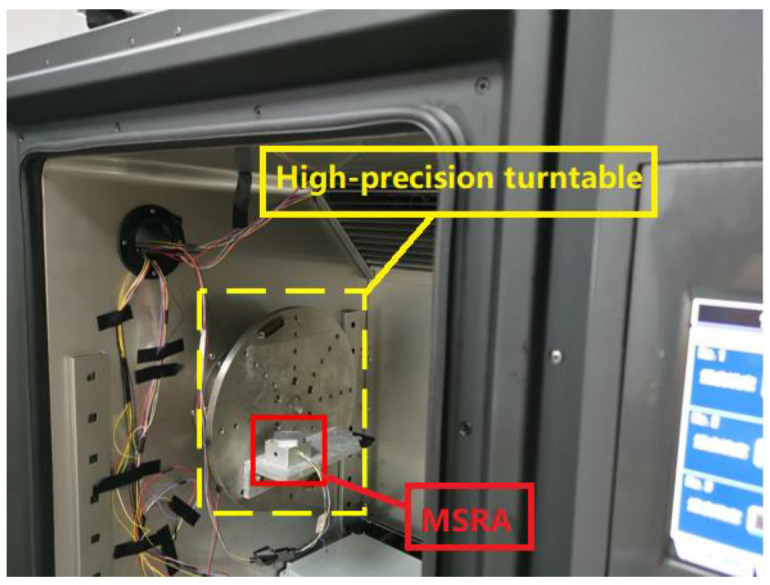
Temperature experimental system.

Determination of the parameters of the neural network model requires numerous accelerometer output data and temperature data for training. Therefore, it was necessary to build a temperature experimental system to conduct a series of temperature experiments on the accelerometer. The temperature experimental system mainly consisted of a high-precision turntable, a temperature chamber and a MSRA prototype. The experimental system is shown in Figure 6.

The MSRA prototype was used to conduct a zero-bias experiment at room temperature and a full-temperature experiment. The output of the accelerometer and the experimental temperature were collected. The zero-bias experiment was conducted at room temperature with a sampling rate of one time per second for at least an hour and a half when the input acceleration was zero gravity (0 g). The operating temperature of the MSRA in the full-temperature experiment was between −40 °C and 60 °C, and the test nodes were established at intervals of 20 °C. After the operating temperature reached the expected value, the temperature was maintained for one hour. Each temperature node collected data for four states of acceleration at +0 g, +1 g, −0 g, and −1 g, and each state was collected for at least 30 s while the time interval for data collection was set to one second. The variations of the scale factor and bias at full temperature were used as the evaluation index in the full-temperature experiment. The variation of the scale factor at full temperature was the standard deviation of the scale factor at different temperature points divided by the mean value. The variation of the bias at full temperature was the standard deviation of the bias at different temperature points, and the bias was calculated as:

(12) $$B_{0}=\frac{U_{+0\;g}-U_{-0\;g}}{U_{+1\;g}-U_{-1\;g}}\cdot g$$

where $U_{+0\;g}$, $U_{+1\;g}$, $U_{-0\;g}$, and $U_{-1\;g}$ are the outputs of the accelerometer at +0 g, +1 g, −0 g, and −1 g, respectively.

### 3.2. Results and Discussion

By repeating the zero-bias experiment at room temperature and full temperature experiment, six datasets for the zero-bias experiment at room temperature and five datasets for the full-temperature experiment were obtained, among which four datasets of the zero-bias experiment at room temperature and three datasets of the full-temperature experiment were randomly selected as the training datasets, and the remaining datasets were utilized for evaluation. 

The FA-BP and IFA-BP neural network temperature compensation models used in the experiments have an input layer, a hidden layer, and an output layer. The number of neurons in the input layer is 2, the number of neurons in the hidden layer is 10, and the number of neurons in the output layer is 1. We used the FA-BP model and IFA-BP model to train the datasets 30 times, and the FA-BP model and IFA-BP model with the smallest RMSE (root mean square error) were selected for comparison. To evaluate the performance of the compensation model for different test datasets, the two test datasets of the zero-bias experiment at room temperature were compensated by the same model, and the two test datasets of the full-temperature experiment were also compensated by the same model.

**Figure 7:**
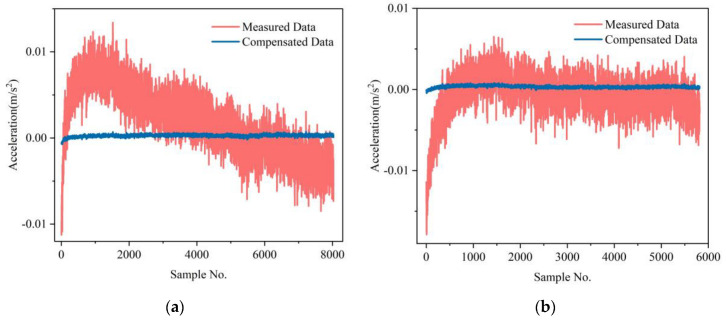
The IFA-BP compensation results of the accelerometer at room temperature: (**a**) test dataset 1; (**b**) test dataset 2.

For the zero-bias experiments at room temperature, the original data indicators of the MSRA and the indicators after compensation by the FA-BP model and IFA-BP model are shown in Table 1. According to the table, it can be seen that the zero-bias stability of the accelerometer has been significantly improved by the neural network, and the same compensation model is effective for different test datasets, indicating the applicability of the temperature compensation model based on a neural network. Among them, after compensation by the IFA-BP model, the zero-bias stability after 30 min of startup, zero-bias stability after 20 min of startup, and zero-start zero-bias stability are better than those of the FA-BP model. A comparison of the MSRA before and after compensation by the IFA-BP is shown in Figure 7. For graphing convenience, a mean value was subtracted from the measured data.

**Figure 8:**
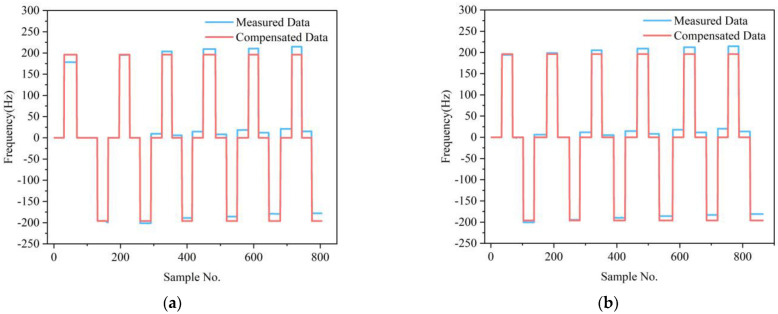
The IFA-BP compensation results of the accelerometer in the full-temperature experiment: (**a**) test dataset 1; (**b**) test dataset 2.

**Figure 9:**
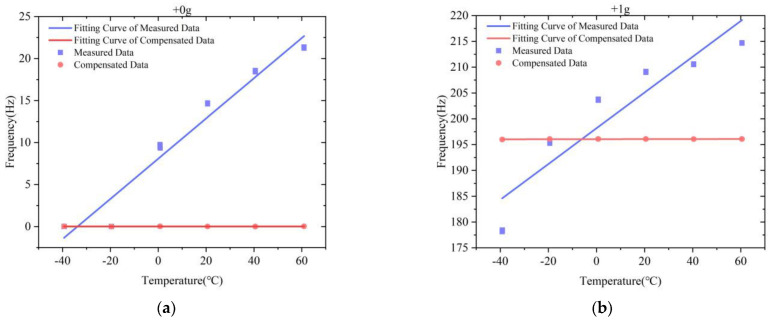

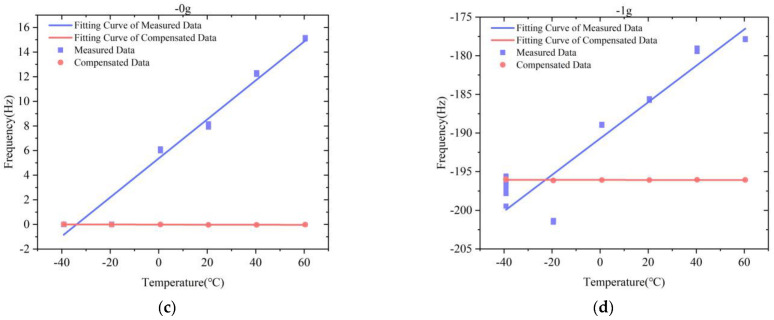
The IFA-BP compensation results of test dataset 1 in the full-temperature experiment: (**a**) +0 g; (**b**) +1 g; (**c**) −0 g; (**d**) −1 g.

**Figure 10:**
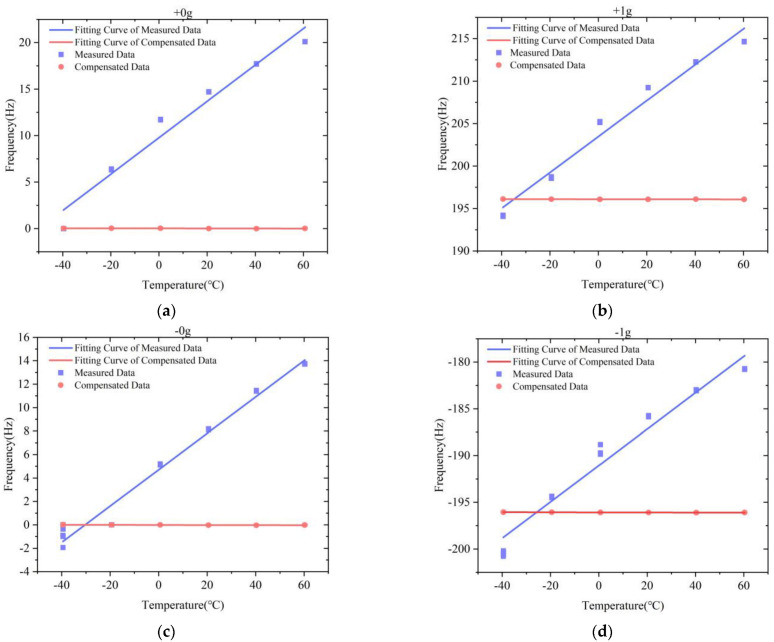
The IFA-BP compensation results of test dataset 2 in the full-temperature experiment: (**a**) +0 g; (**b**) +1 g; (**c**) −0 g; (**d**) −1 g.

The variation of the scale factor and bias at full temperature of two test datasets before and after compensation are shown in Table 2. The comparison results indicate that the IFA-BP model has a better effect in improving the accelerometer’s performance at full temperature than the FA-BP model. The variation of the scale factor at full temperature after compensation by the IFA-BP model has improved by more than 70 times, and the variation of the bias at full temperature has improved by around three orders of magnitude. Figure 8 shows a comparison of the frequency output of the MSRA at six temperature points and four states before and after compensation by the IFA-BP model. Figure 9 and Figure 10 show the curves of the accelerometer’s output with temperature before and after IFA-BP neural network model compensation at four acceleration states. In these figures, it can be seen that the accelerometer’s frequency output before compensation is affected by the temperature and produces frequency drift. After compensation by the IFA-BP model, the temperature performance of accelerometer has improved greatly.

## 4. Conclusions

In order to improve the temperature performance of MRSAs, an accelerometer temperature compensation method based on an improved firefly algorithm optimized BP neural network was proposed in this study. The IFA was used to optimize the initial values of the BP neural network to improve the convergence accuracy and robustness of the neural network’s training. Zero-bias experiments at room temperature and full-temperature experiments were conducted on the MSRA, and temperature compensation models of the FA-BP and IFA-BP neural networks were established. A comparison of the accelerometer’s output before and after compensation shows that the proposed IFA-BP neural network temperature compensation model is effective. The zero-bias stability of the accelerometer in the zero-bias experiment at room temperature improved by more than 10 times. The variation of the scale factor at full temperature improved by more than 70 times and the variation of the bias at full temperature improved by around 1000 times. The results indicate that the temperature compensation method based on the IFA-BP neural network is suitable for MRSAs in both zero-bias experiments at room temperature and full-temperature experiments.

## Figures and Tables

**Table 1:** Zero-bias stability at room temperature before and after compensation.

	Measured Data		FA-BP		IFA-BP	
	Test Dataset 1	Test Dataset 2	Test Dataset 1	Test Dataset 2	Test Dataset 1	Test Dataset 2
Zero-bias stability after 30 min of startup (μg)	186.47	109.56	15.867	14.476	7.7562	7.4809
Zero-bias stability after 20 min of startup (μg)	231.63	136.38	16.902	19.417	9.6671	10.127
Zero-start zero-bias stability (μg)	283.05	227.98	24.848	25.907	11.868	12.750

**Table 2:** The variation of scale factor and bias at full temperature before and after compensation.

	Measured Data		FA-BP		IFA-BP	
	Test Dataset 1	Test Dataset 2	Test Dataset 1	Test Dataset 2	Test Dataset 1	Test Dataset 2
The variation of the scale factor at full temperature (ppm)	20,600	2153.2	578.77	107.59	214.86	30.806
The variation of the bias at full temperature (μg)	39,152	32873	89.431	103.57	32.967	36.556

## References

[1] Huang L., Yang H., Gao Y., Zhao L., Liang J. (2013). Design and implementation of a micromechanical silicon resonant accelerometer. Sensors.

[2] Weinberg M.S., Bernstein J.J., Borenstein J.T., Campbell J., Cousens J., Cunningham R.K., Fields R., Greiff P., Hugh B., Niles L. Micromachining inertial instruments. Proceedings of the Micromachining and Microfabrication Process Technology II.

[3] Hopkins R., Miola J., Sawyer W., Setterlund R., Dow B. The silicon oscillating accelerometer: A high-performance MEMS accelerometer for precision navigation and strategic guidance applications. Proceedings of the Institute of Navigation, 2005 National Technical Meeting, NTM 2005.

[4] Pike W.T., Delahunty A., Mukherjee A., Dou G., Liu H., Calcutt S., Standley I.M. A self-levelling nano-g silicon seismometer. Proceedings of the SENSORS, 2014 IEEE.

[5] Jiang B., Huang S., Zhang J., Su Y. (2020). Analysis of Frequency Drift of Silicon MEMS Resonator with Temperature. Micromachines.

[6] Jing Z., Anping Q., Qin S., You B., Guoming X. Research on temperature compensation method of silicon resonant accelerometer based on integrated temperature measurement resonator. Proceedings of the 2015 12th IEEE International Conference on Electronic Measurement & Instruments (ICEMI).

[7] Kyu Lee H., Melamud R., Kim B., Chandorkar S., Salvia J.C., Kenny T.W. (2013). The effect of the temperature-dependent nonlinearities on the temperature stability of micromechanical resonators. J. Appl. Phys..

[8] Zhang X., Park S., Judy M.W. (2007). Accurate Assessment of Packaging Stress Effects on MEMS Sensors by Measurement and Sensor–Package Interaction Simulations. J. Microelectromechan. Syst..

[9] Luschi L., Iannaccone G., Pieri F. (2017). Temperature Compensation of Silicon Lame Resonators Using Etch Holes: Theory and Design Methodology. IEEE Trans. Ultrason. Ferroelectr. Freq. Control.

[10] Mustafazade A., Seshia A.A. Compact High-Precision Analog Temperature Controller for MEMS Inertial Sensors. Proceedings of the 2018 IEEE International Frequency Control Symposium (IFCS).

[11] Salvia J.C., Melamud R., Chandorkar S.A., Lord S.F., Kenny T.W. (2010). Real-Time Temperature Compensation of MEMS Oscillators Using an Integrated Micro-Oven and a Phase-Locked Loop. J. Microelectromechan. Syst..

[12] Shin D.D., Chen Y., Flader I.B., Kenny T.W. Epitaxially encapsulated resonant accelerometer with an on-chip micro-oven. Proceedings of the 2017 19th International Conference on Solid-State Sensors, Actuators and Microsystems (TRANSDUCERS).

[13] Yang B., Dai B., Liu X., Xu L., Deng Y., Wang X. (2014). The on-chip temperature compensation and temperature control research for the silicon micro-gyroscope. Microsyst. Technol..

[14] Cui J., Yang H., Li D., Song Z., Zhao Q. (2019). A Silicon Resonant Accelerometer Embedded in An Isolation Frame with Stress Relief Anchor. Micromachines.

[15] Kang H., Ruan B., Hao Y., Chang H. (2020). A Mode-Localized Resonant Accelerometer With Self-Temperature Drift Suppression. IEEE Sens. J..

[16] Li H., Huang L., Ran Q., Wang S. Design of Temperature Sensitive Structure for Micromechanical Silicon Resonant Accelerometer. Proceedings of the 2017 International Conference on Computer Network, Electronic and Automation (ICCNEA).

[17] Li N., Xing C., Sun P., Zhu Z. Simulation Analysis on Thermal Drift of MEMS Resonant Accelerometer. Proceedings of the 2019 20th International Conference on Electronic Packaging Technology (ICEPT).

[18] Shin D.D., Ahn C.H., Chen Y., Christensen D.L., Flader I.B., Kenny T.W., IEEE Environmentally Robust Differential Resonant Accelerometer in a Wafer-Scale Encapsulation Process. Proceedings of the 30th IEEE International Conference on Micro Electro Mechanical Systems (MEMS).

[19] Cui J., Liu M., Yang H., Li D., Zhao Q., IEEE Temperature Robust Silicon Resonant Accelerometer with Stress Isolation Frame Mounted on Axis-Symmetrical Anchors. Proceedings of the 33rd IEEE International Conference on Micro Electro Mechanical Systems (MEMS).

[20] Zotov S.A., Simon B.R., Trusov A.A., Shkel A.M. (2015). High Quality Factor Resonant MEMS Accelerometer With Continuous Thermal Compensation. IEEE Sens. J..

[21] Shi R., Zhao J., Qiu A.P., Xia G.M. (2013). Temperature Self-Compensation of Micromechanical Silicon Resonant Accelerometer. Appl. Mech. Mater..

[22] Cai P., Xiong X., Wang K., Wang J., Zou X. (2021). An Improved Difference Temperature Compensation Method for MEMS Resonant Accelerometers. Micromachines.

[23] Araghi G., Landry R., IEEE Temperature compensation model of MEMS inertial sensors based on neural network. Proceedings of the IEEE/ION Position, Location and Navigation Symposium (PLANS).

[24] Cao H., Zhang Y., Shen C., Liu Y., Wang X. (2018). Temperature Energy Influence Compensation for MEMS Vibration Gyroscope Based on RBF NN-GA-KF Method. Shock Vib..

[25] Fontanella R., Accardo D., Lo Moriello R.S., Angrisani L., De Simone D. (2018). MEMS gyros temperature calibration through artificial neural networks. Sens. Actuators A Phys..

[26] Wang S., Zhu W., Shen Y., Ren J., Gu H., Wei X. (2020). Temperature compensation for MEMS resonant accelerometer based on genetic algorithm optimized backpropagation neural network. Sens. Actuators A Phys..

[27] Lu Q., Shen C., Cao H., Shi Y., Liu J. (2019). Fusion Algorithm-Based Temperature Compensation Method for High-G MEMS Accelerometer. Shock Vib..

[28] Zhu M., Pang L., Xiao Z., Shen C., Cao H., Shi Y., Liu J. (2019). Temperature Drift Compensation for High-G MEMS Accelerometer Based on RBF NN Improved Method. Appl. Sci..

[29] Hornik K. (1991). Approximation Capabilities of Multilayer Feedforward Networks. Neural Netw..

[30] Liu M., Zhang M., Zhao W., Song C., Wang D., Li Q., Wang Z. Prediction of congestion degree for optical networks based on bp artificial neural network. Proceedings of the 2017 16th International Conference on Optical Communications and Networks (ICOCN).

[31] Ren C., An N., Wang J., Li L., Hu B., Shang D. (2014). Optimal parameters selection for BP neural network based on particle swarm optimization: A case study of wind speed forecasting. Knowledge-Based Syst..

[32] Yang X.-S. Firefly algorithms for multimodal optimization. Proceedings of the International Symposium on Stochastic Algorithms.

[33] Yang X.-S. (2010). Firefly Algorithm, Lévy Flights and Global Optimization. Research and Development in Intelligent Systems XXVI.

[34] Metropolis N., Rosenbluth A.W., Rosenbluth M.N., Teller A.H., Teller E. (1953). Equation of state calculations by fast computing machines. J. Chem. phys..

